# Targeting Chromatin Complexes in Myeloid Malignancies and Beyond: From Basic Mechanisms to Clinical Innovation

**DOI:** 10.3390/cells9122721

**Published:** 2020-12-21

**Authors:** Florian Perner, Scott A. Armstrong

**Affiliations:** 1 Department of Pediatric Oncology, Dana-Farber Cancer Institute, Harvard Medical School, Boston, MA 02215, USA; scott_armstrong@dfci.harvard.edu; 2 Internal Medicine II, Hematology and Oncology, Friedrich Schiller University Medical Center, 07747 Jena, Germany

**Keywords:** chromatin, EZH2, DOT1L, LSD1, Menin, MLL1, KMT2A, BRD4, BRD9, PPI, PROTAC, epigenetics, transcription, degradation, methylation, acetylation

## Abstract

The aberrant function of chromatin regulatory networks (epigenetics) is a hallmark of cancer promoting oncogenic gene expression. A growing body of evidence suggests that the disruption of specific chromatin-associated protein complexes has therapeutic potential in malignant conditions, particularly those that are driven by aberrant chromatin modifiers. Of note, a number of enzymatic inhibitors that block the catalytic function of histone modifying enzymes have been established and entered clinical trials. Unfortunately, many of these molecules do not have potent single-agent activity. One potential explanation for this phenomenon is the fact that those drugs do not profoundly disrupt the integrity of the aberrant network of multiprotein complexes on chromatin. Recent advances in drug development have led to the establishment of novel inhibitors of protein–protein interactions as well as targeted protein degraders that may provide inroads to longstanding effort to physically disrupt oncogenic multiprotein complexes on chromatin. In this review, we summarize some of the current concepts on the role epigenetic modifiers in malignant chromatin states with a specific focus on myeloid malignancies and recent advances in early-phase clinical trials.

## 1. Histone-Code and Epigenetic Networks: Implications for Myeloid Malignancies and Other Cancer Types

The genomic DNA in eukaryotic cells is wrapped around a core histone octamer composed of Histone H2A (H2A), Histone H2B (H2B), Histone H3 (H3), and Histone H4 (H4) [1,2,3]. This complex of DNA and histones is compacted at different densities to chromatin, ranging from a simple chain of DNA and histones to highly condensed metaphase chromosomes [4,5,6,7,8,9]. The tight regulation of chromatin condensation and de-condensation is vital for a healthy organism since it is crucial for the equal distribution of genetic information to the daughter cells during cell division [10,11]. Furthermore, the state of chromatin condensation and therefore the degree of DNA accessibility, restricts or allows transcription factors and the RNA-polymerase machinery to physically interact with the DNA. Therefore, the stringent regulation of chromatin accessibility is a critical determinant of proper spatial and temporal regulation of gene transcription [12,13,14]. In cancer cells, the healthy chromatin homeostasis is disrupted by a variety of mechanisms promoting aberrant transcription and potentially also cytogenetic alterations [15,16,17].

**Figure 1:**
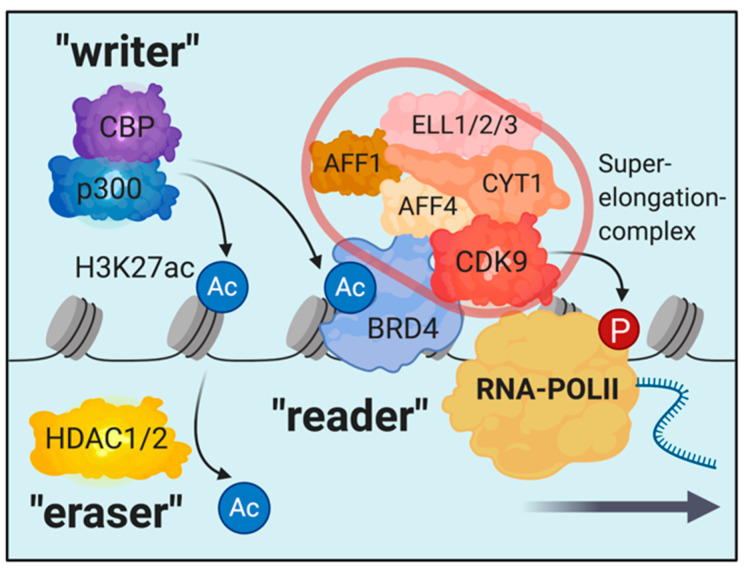
Schematic illustrating the concept of writers, erasers, and readers on the example of the H3K27ac-mark. Multiprotein-complexes are recruited and/or associate with reader proteins for locus specific chromatin localization. In this example, the superelongation-complex, which is required for RNAPolII activation, associates with BRD4.

The dynamics of chromatin biology are regulated by a variety of post-translational modifications at the unstructured core-histone tails [18]. These modifications involve methylation, acetylation, and phosphorylation, as well as many other less well characterized modifications like sumoylation, ADP-ribosylation, deimination, proline-isomerization, and crotonylation [18,19,20,21,22,23]. Early studies in the field have provided evidence that histone acetylation, a mark that is broadly associated with active chromatin, diminishes the positive charge of histones, thereby loosening the interaction between the histones and the negatively charged phosphate groups on the DNA, resulting in increased DNA accessibility [23,24,25,26,27,28,29]. In 2000, Brian Strahl and David Allis suggested a groundbreaking concept by proposing the model of the “histone code” [22]. Several observations from their and other groups prompted the idea that the complex array of post-translational modifications on histone tails serve as a binding platform for reader proteins and associated multiprotein complexes and thereby orchestrate a complex network of regulatory proteins on chromatin [22,30,31,32,33,34]. This concept is the basis for our current understanding of the regulatory dynamics on chromatin involving writers, erasers, and readers of histone modifications (Figure 1). An example is H3K27 acetylation, which is associated with active enhancers and promotors [18]. The “writers” of this mark are the acetyltransferases p300 and CBP that deposit H3K27ac to activate specific enhancers and promotors [35,36]. Histone-deacetylases (like HDAC1 and 2) serve in an antagonistic fashion as “erasers” to remove the mark and repress these regulatory elements. BRD4, that can associate with H3K27ac nucleosomes via its bromodomain, is a “reader” that gets recruited to active promotors and enhancers through binding to H3K27ac [37,38,39,40]. Importantly, BRD4 associates with the multiprotein-complex of the transcription machinery and is essential for the activation of RNA-polymerase II (RNA-PolII) by the superelongation complex [41]. Therefore, altering the function of readers, writers, or erasers in this system will have dramatic consequences for the homeostasis of gene transcription [40,42,43,44,45,46].

In actual physiological systems, the complexity of the protein-network generated by the histone code is complicated, since the regulatory elements of different marks are highly interconnected. Writers and erasers of histone modifications get recruited by reader proteins of other histone modifications, leading to a topologically organized array of co-existing modifications and associated multi-protein complexes that define distinct regulatory microenvironments at specific regions on chromatin impacting transcription and 3D-chromosome architecture [47,48,49,50,51,52]. These regulatory networks are central mediators of embryonic development, lineage determination during differentiation and cellular homeostasis [53,54,55,56,57,58,59,60,61,62,63,64,65]. Consequently, aberrant regulation of this network is a hallmark of cancer development and progression [17,66,67,68,69,70]. Several of the recurrently detected genetic abnormalities in patients with myeloid malignancies involve epigenetic modifiers like DNMT3A, TET2, ASXL1, EZH2, IDH1/2, KMT2A, KAT6A, KDM5A, KDM6A, or NSD1 [71,72,73,74]. In the recent years, it has become clear that mutations in some of these genes arise in hematopoietic stem cells of healthy individuals during the process of aging [75,76,77,78]. The occurrence of these mutations was found to be associated with an increased risk to develop myeloid malignancies as well as cardiovascular disease [75,79,80]. The application of novel single-cell sequencing approaches in primary material from patients with acute myeloid leukemia (AML) and myeloproliferative neoplasia (MPN) revealed that these clonal mutations in epigenetic modifiers tend to co-occur in dominant clones of patients that ultimately developed leukemia, implicating a synergistic pathogenic function if occurring in the same cell. Mutations in signaling molecules, on the other hand, appeared to originate later during disease evolution and showed a tendency not to co-occur within the same clone [81]. These recent findings strongly suggest, that epigenetic rewiring in specific hematopoietic clones is the foundation for the development of myeloid malignancies and may explain the increasing incidence of myelodysplastic syndrome (MDS), MPN, and AML in elderly individuals. Nevertheless, no pharmacological targeting approaches that show promise in blocking expansion and molecular progression of these pre-leukemic clones have been established yet clinically. Gaining a better understanding of the chromatin biology during this preleukemic disease state and developing valid predictors of progression, as well as clinically active epigenetic mono- or combination therapies, will be a central goal to prevent leukemia development in the future. However, much progress has been made in the preclinical and clinical development of compounds interfering with the function of chromatin modifiers for use in patients with fully developed cancer. In both myeloid malignancies and solid cancers, several of these molecules have entered clinical trials and show activity in a variety of diseases [74,82,83,84,85]. In this review, we will discuss several clinical and pre-clinical epigenetic targeting approaches for cancer therapy in the context of the molecular mechanisms of action of different drug-classes and their potential to disrupt oncogenic epigenetic signatures. We will particularly focus on therapeutic tools that target specific chromatin complexes and will not discuss the clinical use of inhibitors of DNA methylation. The demethylating agents 5-azacitidine and decitabine were the first “epigenetic” drugs that were approved for the treatment of MDS and AML by the US Food and Drug Administration (FDA) in 2004 and are successfully used in different clinical settings to date [84]. Nevertheless, their exact mechanism of action is not fully understood and they have been previously reviewed [82,83,84]. Therefore, we will not discuss these compounds in further detail in this review.

## 2. Inhibition of Histone Methylation on Chromatin: Recent Clinical Advances and Challenges

Targeting histone-methylation pattern on chromatin is a scientifically and clinically exiting opportunity to interrogate with disease-related epigenetic programs in vitro and in vivo. Given the fact, that a very specific set of methyltransferases and de-methylases is responsible for the deposition and removal of specific methylation-marks, the interrogation with distinct de-regulated pattern within the histone-code is possible. In contrast, histone acetyltransferases and deacetylases act in a more promiscuous way. Those enzymes have a very broad spectrum of targets among histones as well as non-histone proteins. Therefore, the molecular consequences of inhibiting those enzymes are more complex and much less specific for certain epigenetic programs. Multiple inhibitory drugs for methyltransferases, acetyltransferases, demethylases, and deacetylases have been developed and are currently under preclinical or clinical investigation [83]. In the history of drug-discovery, much experience has been gathered regarding the development and evaluation of enzymatic inhibitors [86,87,88]. Those drugs act as competitive or allosteric inhibitors of catalytically active proteins, preventing the binding of an enzyme to its target substrate and/or disturbing the structural integrity of its catalytic center. In the epigenetic space, proteins with enzymatic activity play a crucial role as “writers” and “erasers” of histone modifications, as discussed above. Therefore, molecules that inhibit the activity of those proteins have the potential to specifically interfere with the global deposition of certain histone-marks. A number of these molecules have been developed for the treatment of hematologic malignancies and solid cancers [83]. The first inhibitors of histone modifiers that have been approved for the treatment of cancer were inhibitors of histone-deacetylases. The first-in class molecule Vorinostat was initially approved for cutaneous T cell lymphoma [89]. Drug development efforts to improve the pharmacologic properties led to the approval of Belinostat in 2014 for T-cell lymphoma and Panobinostat in 2015 for refractory multiple myeloma. In myeloid malignancies, HDAC inhibitors show promise in clinical trials as monotherapy or in combination with other drugs [83,90,91,92]. Using this class of drugs for the precise interrogation of specific epigenetic programs/mechanisms has however remained difficult. As mentioned above, HDACs act in a promiscuous way erasing histone acetylation broadly across the genome. Furthermore, clinically active compounds are mostly pan-HDAC-inhibitors without particular selectivity for a specific molecule. Therefore, distinct oncogenic signatures or somatic mutations that confer sensitivity to HDAC-inhibition have not been established. More recently, the evolving knowledge about epigenetic mechanisms and subsequent drug development efforts have led to the establishment of a number of inhibitors of histone-methyltransferases. Histone methyltransferases appear to act in a more selective fashion than acetyltransferases. This class of compounds may enable scientists and clinicians for the first time to develop mechanistically driven and relatively well tolerated treatment regimens for cancer therapy. In this review, we will focus on specific inhibitors of histone-modifying complexes that have recently entered clinical trials (Table 1).

### 2.1. Inhibition of EZH2—The Catalytic Subunit of the PRC2-Complex

**Figure 2:**
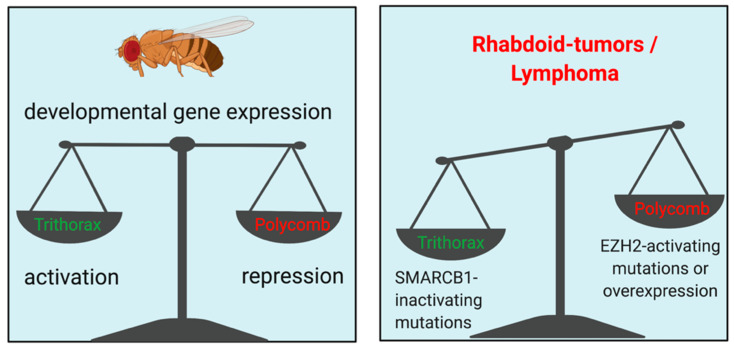
Illustration of the balance between polycomb-mediated repression and trithorax-mediated activation of developmental gene expression programs. Embryonic development and cellular differentiation are regulated by a fine balance between activating and repressive mediators of gene expression and chromatin state (**left**). In rhabdoid tumors and certain lymphomas, this balance is shifted towards a dominance of the polycomb-repressive complex (**right**).

The tight regulation of the interplay between activating and repressive mediators on chromatin is crucial for the determination of cell identity during embryonic development and differentiation and also a critical element of aberrant chromatin biology in cancer cells. The competition between activating Trithorax-group proteins and repressive Polycomb complexes on central developmental genes (e.g., *Hox*-cluster genes) and the importance of this balance for segmental identity has been defined in Drosophila melanogaster [55,57,93,94] (Figure 2). The importance of the interplay between proteins associated with homologues of these chromatin-regulator families is highly conserved from yeast to mammalian cells and de-regulation of this balance is frequently observed in cancer cells [55,57,95,96,97,98,99,100,101].

One of the histone modifications that is associated with polycomb (PRC)-mediated repression is H3K27me3. This modification is catalyzed by the histone methyltransferase “Enhancer of zeste 2” (EZH2), which is the catalytic subunit of the PRC2-complex [102]. H3K27me3 is strongly associated with repressed chromatin and is crucial for the repression of developmental programs in a timely manner [103,104,105]. Mutations and aberrant expression of EZH2 are frequently found in cancer and targeting this methyltransferase may have therapeutic potential in different disease entities [106,107,108,109,110,111,112]. Based on pre-clinical data and the early experiences from clinical trials, sensitivity to EZH2-inhibition seems to be determined by molecular settings that result in a specific dependency on PRC2 function. Activating mutations in EZH2 are frequently found in lymphoma and are considered disease drivers that display a target for pharmacological inhibition [110,113,114,115]. Overexpression of EZH2 is observed in a variety of different cancers, suggesting potential therapeutic opportunities [111,112,115,116]. Furthermore, specific mutations in other chromatin modifying complexes have been shown to create synthetic-lethality with EZH2-inhibition. As mentioned above, the interplay between activating trithorax-group proteins and PCR-complexes is crucial for the regulation of developmental genes (Figure 2, left). Inactivating mutations within members of the SWI/SNF chromatin remodeling complexes, as members of the trithorax-group, lead to a shift of this balance towards the dominance of PRC-mediated repression (Figure 2, right). SWI/SNF complex alterations are among the most frequently found genetic alterations across all cancer types and are disease drivers in a variety of entities, most intensively characterized in rhabdoid sarcoma [117,118,119,120,121]. In rhabdoid tumors with SMARCB1 loss-of-function mutations, EZH2 displays a specific dependency which can be exploited using pharmacologic inhibition of EZH2′s enzymatic function [122,123]. In myeloid malignancies, the relevance of altered EZH2 function largely remains elusive. In MPN, MDS and AML, mutations of EZH2 and other members of the PRC2 complex are as recurrent events. Of note, the vast majority of these mutations confer a loss of PRC2 function [124,125,126]. In contrast to the observations in sarcoma, EZH2 seems to act as a tumor suppressor in these myeloid conditions, since its inactivation by somatic mutations is associated with adverse outcomes [127,128,129,130]. Conversely, homologues to drosophila’s trithorax-family like the histone methyltransferase MLL1 (KMT2A) and certain SWI/SNF complex members appear to act as oncogenes/dependencies in myeloid malignancies, suggesting fundamentally different consequences of the trithorax–polycomb balance in myeloid neoplasms compared to rhabdoid- and other tumors [131,132,133]. Therefore, targeting of EZH2 using pharmacologic inhibitors does not appear to be intuitive in these conditions. Nevertheless, specific molecular constellations and/or the exploitation of so far unknown synthetic lethalities might allow the use of these drugs in certain settings in the future. For example, it has been demonstrated that chronic myeloid leukemia (CML) stem cells rely on EZH2 for self-renewal and disease maintenance and that EZH2 inhibition in combination with targeting of BCR-ABL can be utilized to eradicate these disease-initiating cell populations in a pre-clinical setting [134,135]. Furthermore, the PRC2 complex appears to be required for proliferation and BET-inhibitor resistance in *MLL*-rearranged AML models [136,137]. More detailed molecular insights into the chromatin biology of these diseases, particularly in the disease-driving cell populations, are required to evaluate the potential of pharmacologic inhibition of the PRC2-repressive complex in myeloid conditions.

**Figure 3:**
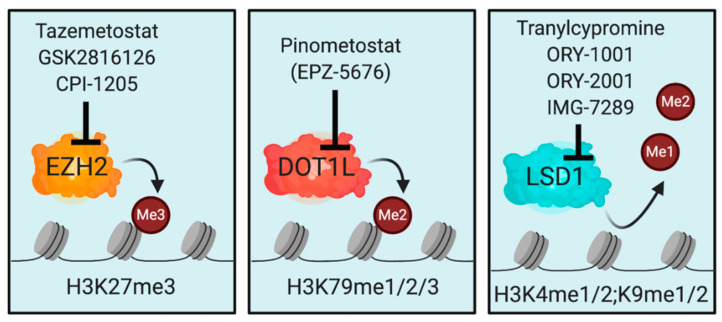
Enzymatic inhibitors of epigenetic writers and readers in clinical trials. Illustration of the targets of EZH2, DOT1L, and LSD1-inhibition with annotation of compounds that are approved by the FDA or entered clinical trials.

Tazemetostat, a selective inhibitor of EZH2, was the first compound of this class that was approved by the FDA for the treatment of locally advanced epitheloid sarcoma and follicular lymphoma in 2020 [138,139] (Figure 3). The response rate in SMARCB1-negative epitheloid sarcoma was modest with an overall response rate of 15%, while only 1.6% of patients reached a complete remission. Most responses lasted 6 months or longer and the drug was well tolerated [139]. Due to the lack of therapeutic alternatives, Tazemetostat was approved based on this phase 2 clinical trial in January 2020. In relapsed/refractory follicular lymphoma harboring activating mutations of EZH2, the clinical responses were much more encouraging, with an overall response rate of 69% and 12% complete responses [138]. The results of this clinical trial led to approval of Tazemetostat for relapsed/refractory follicular lymphoma with EZH2 mutations in June 2020. A number of clinical trials evaluating Tazemetostat in different clinical settings in lymphoma and solid tumors are currently ongoing [83,111,140]. GSK2816126, another EZH2-inhibitor, was recently investigated in a phase 1 clinical trial in lymphoma and solid cancers. The drug was well tolerated, but failed to potently repress H3K27me3, as measured in the peripheral blood of patients potentially due to unfavorable pharmacologic properties [141]. The second-generation compound CPI-1205 that has improved potency for inhibition of EZH2 is currently investigated in phase 1–2 clinical trials in lymphoma and solid cancers [142,143]. Data from these trials have not been released.

### 2.2. Inhibition of the Histone Methyltransferase DOT1L in MLL-Rearranged Leukemia and Beyond

DOT1L is the only histone-methyltransferase that catalyzes H3K79me1, -me2, and -me3 (Figure 3). H3K79me is deposited across the gene body and is associated with transcriptionally active genes [18,144,145,146]. In adult mammalian organisms, DOT1L is dispensable for homeostasis of most cell types except hematopoietic stem cells [147,148]. MLL1 (KMT2A), which is the mammalian homologue of Drosophila trithorax, is recurrently affected by chromosomal translocations during the development of acute leukemia, leading to the generation of oncogenic fusion proteins [149,150,151,152]. These MLL1-fusion oncogenes recruit DOT1L directly via the fusion partner (e.g., AF9, AF10, AF17, or ENL) or indirectly to their target genes to establish an active chromatin environment and prevent repression [153,154,155,156,157,158,159,160,161,162]. Consequently, genetic inactivation or pharmacologic inhibition of DOT1L has been established as a tool to target the MLL-fusion driven transcriptional program [154,163,164,165,166,167]. Of note, other subtypes of leukemia that show a high dependency on wild-type MLL1, like NPM1-, DNMT3A-, and IDH1/2-mutated as well as NUP98-rearranged AML, have been shown to be likewise sensitive to inhibition of DOT1L [153,168,169,170,171]. Given the fact that DOT1L is not required in normal tissues, pharmacological inhibition of DOT1L could be a highly selective treatment strategy in *MLL*-rearranged and MLL-dependent leukemia. EPZ004777, the first selective DOT1L inhibitor reported in 2011, led to a potent reduction in H3K79me and cell proliferation in the micromolar range [167]. In an initial in vivo assessment using a cell-line xenograft system, the drug showed only modest activity due to poor pharmacologic properties. The second-generation DOT1L-inhibitor EPZ5676 showed a higher potency and selectivity to inhibit DOT1L with activity in the low nanomolar range in vitro [166]. Nevertheless, also EPZ5676 showed relatively poor pharmacologic properties in vivo and had to be administrated via continuous infusion to achieve relevant plasma concentrations and clinical responses. In a rat cell-line xenograft model, EPZ5676 led to complete regression of tumors after 3 weeks of administration while no relevant signs of toxicity were reported from this pre-clinical trial [166]. Recent efforts in structure-guided probe optimization have led to the establishment of a set of DOT1L-inhibitors with improved pharmacologic properties including attenuated plasma concentrations and oral bioavailability that were recently reported to have promising pre-clinical activity in patient-derived xenograft models of AML [172,173]. The availability of these novel compounds enables pre-clinical investigators for the first time to probe combination therapies of DOT1L-inhibitors with other epigenetic compounds using clinically applicable dosing regimens in animal model systems. Collectively, these pre-clinical reports provided proof that pharmacological targeting of DOT1L could be an effective and selective treatment approach in *MLL*-rearranged leukemia. However, they also pointed out difficulties regarding DOT1L as a drug target: Both the in vitro and in vivo experiences showed that only complete and prolonged inhibition of DOT1L and consequently H3K79me was sufficient to induce relevant therapeutic responses. The sub-optimal pharmacological properties of both EPZ004777 and EPZ5676 may therefore be problematic.

Despite these pharmacologic limitations, EPZ5676 was investigated as a single-agent in a phase 1 clinical trial in relapsed/refractory MLL-rearranged AML (NCT01684150) [165]. In this study, patients with AML and ALL, 39 of which had *MLL*-rearranged leukemia, were enrolled in 6 dose-escalation and 2 expansion cohorts. Similar to the pre-clinical settings, EPZ5676 (Pinometostat) was administrated via continuous infusion over 28 days. Steady-state plasma concentration was reached after 4–8 h after start of the infusion but decreased rapidly upon discontinuation. Overall, the treatment was well tolerated and a maximum tolerated dose was not reached in part due to physical limitations in solubility and infusion rate of the drug. Out of the 51 patients that received the study medication, only 3 patients completed the study. The main reason for discontinuation was insufficient response and disease progression (31 patients, 60%). Two patients experienced a complete remission on the study medication but ultimately relapsed shortly after the last cycle of EPZ5676. In summary, the trial provided proof of concept for the tolerability and clinical activity of DOT1L-inhibition in *MLL*-rearranged leukemia. Nevertheless, efficacy of the treatment as a monotherapy was limited, inducing modest responses that did not last [165]. Currently, EPZ5676 is under active investigation in two clinical trials, testing its efficacy in relapsed and therapy refractory AML patients with *MLL*-rearrangements in combination with 5-azacitidine (NCT03701295) and in patients with newly diagnosed *MLL*-rearranged AML in combination with standard induction chemotherapy (NCT03724084). Both trials are still recruiting participants. In summary, DOT1L-inhibition may be a useful pharmacological opportunity in myeloid malignancies and potentially also in other cancers. However, to achieve sufficient clinical responses, the establishment of compounds with more favorable pharmacological properties and synergistic combination therapy regimens are likely necessary.

### 2.3. Inhibition of LSD1 in Myeloid Leukemia and Beyond

The lysine-specific demethylase 1 (LSD1) has been identified as a critical eraser for H3K9me1/2 and most importantly for H3K4me1/2 [174,175] on chromatin. H3K4me1 marks enhancers and its tight regulation during cellular differentiation is critical to execute lineage switches and other cell fate decisions [18]. K3K4me1 is catalyzed by MLL3 and MLL4 (KMT2C, KMT2D), a group of trithorax-group proteins that are crucial for shaping enhancers. LSD1 antagonizes this process by demethylation of H3K4 and is therefore able to suppress previously primed enhancers [175,176]. Of note, the demethylase function of LSD1 is specific for mono- and dimethylated H3K4 and H3K9 and has therefore no direct impact on active promotors (H3K4me3) or the heterochromatin (H3K9me3). In embryonic stem cells, it has been shown that loss of LSD1 prevents terminal differentiation, since LSD1 mediated decommissioning of stem-cell enhancers is essential to license lineage-specific rearrangement of the enhancer landscape and associated transcriptional programs [176,177]. In cancer cells on the other hand, LSD1 seems to be a gatekeeper of cancer stemness in different malignant entities, and therefore, a therapeutic target for differentiation therapy [178,179]. A possible explanation for these contrary roles in embryonic stem- and cancer cells might be the fact that cancer cells already underwent lineage priming but actively repress enhancers that drive essential genes for terminal differentiation via LSD1. This epigenetic block of cellular differentiation programs can be released by pharmacologic inhibition of LSD1. Evidence suggests that LSD1-inhibition in AML drives differentiation by disrupting stemness-signatures and at the same time, recommissions PU.1 and C/EBPa-dependent enhancers to facilitate myeloid differentiation [180]. The ability of LSD1-inhibitors to drive differentiation in AML has been well described in several different molecular subtypes to date [181,182,183,184,185,186,187]. In solid cancers, LSD1 activity has been reported to be linked to metastasis, cell proliferation, and inhibition of p53 [188,189,190,191].

Tranylcypromine (TCP) was initially approved by the FDA in 1961 as a MAO-inhibitor for mood and anxiety disorders but recently identified as the first inhibitor of LSD1 demethylase function [192]. TCP and other more selective and potent LSD1-inhibitors like ORY-1001, ORY-2001, or IMG-7289 are currently investigated in a number of early phase clinical trials in different conditions, including AML, MPN, MDS, solid cancers, Alzheimer’s, and multiple sclerosis [83,186] (Figure 3). As a single agent, LSD1-inhibitors were able to induce differentiation of AML blasts and control disease burden in a phase 1 clinical trial [193]. Even though monotherapy with highly potent LSD1-inhibitors shows modest activity, synergistic combination therapies seem to be a more promising strategy to address aggressive malignancies like AML. The potential of LSD1-inhibition to sensitize AML-cells to other differentiation-inducing agents, like ATRA, has been recognized in pre-clinical settings as well as in an initial clinical trial [194,195]. Several other early-phase clinical trials probing combinations of LSD1-inhibitors with ATRA or conventional chemotherapy in AML are currently ongoing and have not been published yet [186]. In pre-leukemic conditions, including MPN and MDS, preclinical and emerging clinical data suggests that LSD1-inhibition can control clonal burden and potentially impact disease progression [196,197]. Clinical trials probing the activity of LSD1-inhibition in MDS and MPN are ongoing [186].

## 3. Disrupting Protein-Protein Interactions to Target Epigenetic Complexes

Despite the recent advances in the development and clinical establishment of enzymatic inhibitors of chromatin modifiers, major challenges remain on the way to development of more clinically efficacious drugs that target chromatin complexes. As described above, most enzymatic inhibitors of histone-modifying enzymes that have been assessed to date show only modest clinical activity, particularly when administrated as single agents. Several of these compounds showed sufficient on-target activity in vivo (as measured by global reduction of histone methylation) and were relatively well tolerated. Still, the clinical responses observed were not as extensive as might have been predicted based on the phenotypes described in preclinical model systems using genetic-inactivation and small molecule inhibitors. In recent years, a growing body of evidence suggests that inhibition of any single enzymatic activity/function in a chromatin complex may be insufficient to perturb epigenetic homeostasis and control gene expression in cancers cells. This has recently been demonstrated for complexes containing EZH2 and LSD1 [198,199,200]. Similarly, pharmacologic inhibition of DOT1L’s methyltransferase function shows modest and delayed responses in vitro and in vivo when compared to genetic inactivation that demonstrated DOT1L’s essential function in *MLL*-rearranged leukemia [147,154,164,172,201,202,203,204]. Therefore, we expect approaches that disrupt chromatin associated complexes more extensively may be more therapeutically efficacious.

Another major issue is that only a relatively small fraction of essential proteins on chromatin have enzymatic activity, so the spectrum of potential applications for catalytic inhibitors is naturally limited. Recent advances in the development of inhibitors of protein–protein interactions open up a novel field of potential applications, particularly in the epigenetic space, were a large proportion of the dense network of multiprotein-complexes cannot be pharmacologically addressed using enzymatic inhibitors. In the past few years, a variety of compounds disrupting epigenetic protein–protein interactions have been synthesized and established mostly in vitro [205]. In this section, we will focus on Menin-MLL inhibitors and bromodomain-inhibitors since these molecules have already been intensively investigated in preclinical studies and entered early-phase clinical trials.

### 3.1. Menin-MLL1 Interaction Inhibitors: Novel Compounds with Disruptive Potential

As described above, gain-of-function of the mammalian trithorax-homologue MLL1 (KMT2A) is a recurring pattern in cancer, particularly in acute leukemia. The increased activation of MLL1-target genes, which are part of an evolutionary conserved stem-cell program, is driven by MLL-fusion oncogenes or aberrantly activated WT-MLL1-complexes [149,169,206,207,208]. Menin is an adaptor protein that binds to the N-terminal proportion of MLL1 and is important for MLL1 function [209,210,211]. Therefore, genetic inactivation of Menin or disruption of the Menin-MLL1-interaction has the potential to disturb the integrity of oncogenic MLL1-chromatin complexes [168,169,206,209,212,213,214,215,216,217]. In 2012, the first potent and selective menin-MLL1 interaction inhibitor, MI2-2, was reported to reduce cellular proliferation and oncogenic gene expression in *MLL*-rearranged leukemia [217,218]. Further structural optimization of this lead compound led to the development of MI-503 and MI-463, two highly potent and orally bioavailable compounds with pre-clinical activity against cell-line xenografts of *MLL*-rearranged leukemia [216]. Moreover, it has been demonstrated that the MLL1-menin-interaction is a central vulnerability in NPM1c-mutated leukemia, a much more frequent subtype of AML in adult patients [168]. In this type of leukemia, an intact MLL1-Menin interaction was shown to be required for the expression of critical stem cell-associated genes and could be successfully targeted using MI-503 [168]. Recently, a further improved but structurally related molecule, MI-3454, was reported to have potent effects on proliferation and gene expression in human leukemia cell lines and primary patient leukemia cells. Importantly, MI-3454 induced potent and long-lasting responses in patient-derived xenografts of *MLL*-rearranged and NPM1c mutated leukemia [213]. Recent work has also demonstrated that a different structurally unrelated highly specific inhibitor of the MLL1-menin interaction, VTP50469, is active in the low nanomolar range and suppressed MLL-target gene expression and cell proliferation in models of *MLL*-rearranged leukemia. In a large panel of patient derived xenografts, VTP50469 induced long-lasting complete responses and eradicated disease in a number of *MLL*-rearranged AML- and ALL-grafts [206]. In a model of NPM1c-mutated AML, the same molecule could be utilized intercept AML development and ultimately prevent manifestation of the disease in mice. Additionally, VTP50469 could also be used to target leukemia after disease onset [169]. In both studies, the expression of *HOXA*-cluster genes was not- or only slightly affected by treatment with the inhibitor. The expression of *MEIS1* and *PBX3*, on the other hand, was rapidly and dramatically affected by VTP50469 treatment serving as molecular markers of response, similar to the observations made under treatment of MI-3454 [169,206,213]. ChIP-sequencing studies investigating chromatin occupancy of MLL-fusion complex members at MLL-target genes have shed light on the mechanisms behind this differential response at different loci. Treatment with the Menin-inhibitor VTP50469 leads to a global loss of Menin and DOT1L from chromatin [206], thereby disrupting the integrity of the oncogenic MLL-complex. Importantly, at the highly responsive target genes (MEIS1, PBX3, etc.) VTP50469 treatment also leads to a loss of the MLL-fusion protein itself [169,206]. Therefore, the ability of a Menin-inhibitor to displace MLL1 from its target genes seems to be the best predictor of the impact of the drug on gene expression.

**Figure 4:**
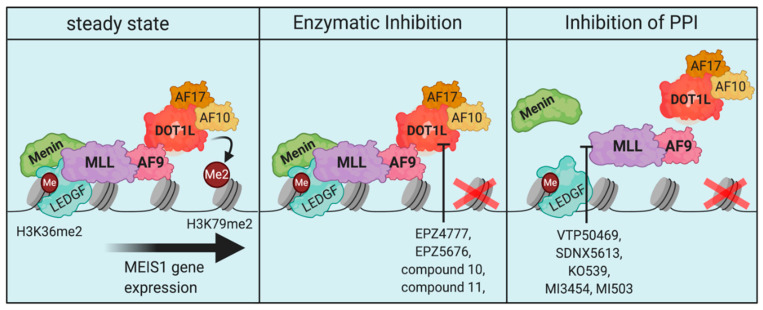
Schematic comparison between the consequences of DOT1L- and Menin-inhibition for the integrity of the MLL-AF9 complex on chromatin. The intact MLL-AF9 complex stably binds to its target genes via Menin and recruits the DOT1L-complex to drive H3K79me2 and gene expression of canonical targets (e.g., MEIS1) (**left**). DOT1L-inhibition removes H3K79me2 by blocking the enzymatic activity of the methyltransferase, while leaving the complex largely intact (**middle**). Inhibition of the menin-MLL1 interaction disrupts the integrity of the MLL-AF9 complex on chromatin, leading to a more rapid and profound response (**right**).

These observations point out, that the potency of pharmacologic inhibition of the menin-MLL1 interaction at certain target genes is critically determined by its ability to physically disrupt the oncogenic multiprotein complex. Along these lines, it is tempting to speculate if this might also be the reason why this class of drugs appears to be more potent than inhibitors of DOT1Ls methyltransferase function. Besides the superior pharmacological features, particularly of VTP50469, MI-503, and MI-3454, their ability to structurally disrupt the oncogenic MLL-complex distinguishes their mechanism of action from DOT1L-inhibition (Figure 4). Using VTP50469, it was demonstrated that the cellular effects induced by this drug are not only more dramatic, but also more rapid compared to EPZ5676 in vitro [206]. Presumably, these features of Menin-inhibitors help to prevent early adaptation of leukemia cells to the drug by not giving them the time they need to adapt their programs. Therefore, Menin-inhibitors are currently a particularly promising group of compounds for selective, potent, and minimally toxic targeted therapy in *MLL*-rearranged and *NPM1c*-mutated leukemia and other MLL1-dependent dependent cancer types.

In November 2019, the AUGMENT-101 trial started to enroll patients on the Menin-inhibitor SDNX-5613 (NCT04065399), which is a close analogue of VTP-50469 [214]. This phase 1/2 clinical trial will enroll 156 participants with relapsed or refractory *MLL*-rearranged and *NPM1c*-mutated leukemia on the study medication. In an early report at the virtual AACR meeting 2020, Syndax pharmaceuticals reported on the first responses observed in patients treated with SDNX-5613. Importantly, the study medication was well tolerated at the first steps of dose-escalation and no dose-limiting toxicities were observed. Two out of three of the patients with *MLL*-rearranged leukemia that were enrolled in the trial showed a response after 28 days of treatment. Remarkably, one of these patients achieved a complete remission irrespective of a low daily drug dose, presumably due to co-medication with CYP3A4 inhibitor. The second patient achieved a partial response after the first cycle of treatment and was continued on the study medication. Treatment of the third patient with *MLL*-rearranged leukemia was discontinued as a result of disease progression at insufficient plasma concentrations of the drug [214]. Another phase 1 clinical trial (KOMET) investigating Kura Oncology’s compound KO-539 (an analogue of MI-3454) is also currently recruiting patients with relapsed/refractory AML (NCT04067336) and recently reported results from the first 6 patients treated, including 2 complete responses [219].

In summary, Menin-MLL interaction inhibitors are currently among the most promising epigenetic compounds under clinical investigation for the treatment of AML with impressive single-agent activity in pre-clinical trials. Their potential to disrupt oncogenic MLL-complexes in contrast to the isolated inhibition of the catalytic activity of DOT1L is presumably one important reason for the increased potency of this therapeutic approach.

### 3.2. Bromodomain-Inhibition: Breaking Apart Acetyl-Reader-Complexes

**Figure 5:**
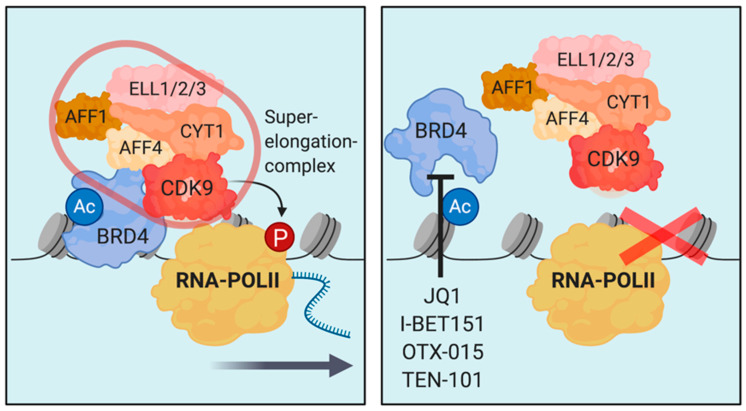
Illustration of the disruption of transcriptional elongation by bromodomain-inhibitors of BRD4. BRD4 binds K3K27ac and associates with the superelongation-complex (SEC) to license phosphorylation and activation of RNA-polymerase II to drive productive transcriptional elongation (**left**). Bromodomain-inhibition of BRD4 prevents binding of BRD4 to H3K27ac preventing association with the SEC and activation of transcription (**right**).

As discussed above, pharmacologic targeting of specific acetylation patterns is challenging due to the fact that acetyltransferases and deacteylases are generally promiscuous, so the interrogation with the deposition of distinct marks is difficult. Bromodomain-containing proteins are readers of acetylated histones, which are part of several different multiprotein complexes that are guided to certain sites on chromatin by these proteins [220,221]. Distinct structural differences within the bromodomains of different proteins of this family enabled the design and synthesis of a large set of fairly specific small molecule inhibitors of protein–protein interactions between acetylated histones and bromodomain proteins [44,220,221,222]. The first bromodomain-inhibitors that proceeded into clinical development were compounds targeting the bromodomains of bromodomain-containing protein 4 (BRD4) and BRD2. BRD4 binds to acetylated H3K27 at active enhancers and super-enhancers and is crucial for transcriptional co-activation, particularly in developmental and oncogenic contexts [37,38,39,107]. In more detail, BRD4 aids in recruitment and activation P-TEFb (CDK9/cyclinT1), thereby controlling transcriptional elongation [41,222] (Figure 5). Efforts to target transcriptional co-activation by BRD4 led to the discovery of the first effective bromodomain-inhibitors, I-BET and JQ1, in 2010 [44,223]. Despite a certain degree of general toxicity, at low doses, BET-inhibitors are fairly selective in inhibiting oncogenic programs, in part by disrupting super-enhancer function [40]. Furthermore, BRD4 was identified as a genetic vulnerability in AML using unbiased screening approaches [43,224]. In these studies, genetic- (RNAi) and pharmacologic inhibition of Brd4 induced differentiation and reduced disease burden in mouse models of *MLL*-rearranged leukemia. Both of these studies found reduced gene expression of MYC as a consequence of enhancer silencing by BET-inhibition as a major factor contributing to the therapeutic efficacy of JQ1 and I-BET151, respectively [43,224]. This phenomenon might be an explanation for the relatively broad, yet fairly selective activity of BET-inhibitors in cancer. Several clinical trials have investigated BET-inhibitors for the treatment of advanced cancers [225]. OTX015 was investigated in a number of clinical trials in hematologic and non-hematologic cancers (NCT02698176, NCT02698189, NCT02259114, NCT01713582, NCT02296476). A common issue in these early phase clinical trials was the fact that dose-limiting toxicities were observed at doses that were not sufficient to induce remission or stable disease and required an intermittent dosing regimen to manage adverse effects [226]. Nevertheless, oral bioavailability and reasonable pharmacokinetics of OTX015 were demonstrated in these trials. The structurally related yet improved compound TEN-101 showed superior pharmacokinetics and first clinical efficacy in NUT-midline carcinoma [227]. A number of clinical trials investigating TEN-101 in AML, myelodysplastic syndromes, multiple myeloma, high-grade B-cell lymphoma, and solid tumors have recently been completed (NCT02308761, NCT03068351, NCT01987362, NCT03292172, NCT03255096). Detailed results from most of these trials are not published yet. Several other BET-inhibitors are currently in clinical- and preclinical development for different malignant conditions [225,228]. Nevertheless, the apparently narrow therapeutic window along with the modest single-agent clinical efficacy of BRD4/2 (and pan-BRD) inhibitors have moderated clinical expectations.

## 4. Hijacking the Proteasome for Targeted Protein Degradation

In the recent years, targeted protein degradation has evolved as a novel and emerging strategy in drug development [229,230,231]. Two major concepts to achieve targeted protein degradation have been established and are currently exploited in drug discovery:

*“Immunomodulatory drugs”* like the FDA-approved compounds Thalidomide, Lenalidomide, and Pomalidomide exert their therapeutic function by selective degradation of a variety of zinc-finger transcription factors, including IKZF1 and IKZF3. Those molecules act as a *“molecular glue”* that binds to its target and subsequently recruits a cereblon E3-ligase complex, leading to polyubiquitination and proteasomal degradation of the target [232,233]. Recent advances in drug discovery strategies have led to screening approaches that allow to exploit this molecular glue concept to design degraders that specifically target other target proteins, opening up the opportunity to address a variety of drug targets including epigenetic modifiers in the future [234,235].

*PROteolysis TArgeting Chimeras* (*PROTACs*) are bifunctional molecules high affinity ligands of ubiquitin ligases like cereblon (CRBN), Von Hippel-Lindau (VHL), or DCAF16 are linked to a highly specific binder to the protein of interest [230,231]. The design and application of PROTACs is rapidly evolving in part due to the fact that highly selective binders of target protein are available for a variety of target proteins in the form of established small molecules that bind to enzymatic pockets or other protein domains. PROTACs successfully targeting kinases, nuclear receptors, and epigenetic modulators have recently been established [230].

In the space of bromodomain-targeting, the differences between bromodomain-inhibition and targeted bromodomain-protein-degradation have been demonstrated and exemplify the potential of this novel targeting approach. In contrast to BET-competitive inhibition, targeted degradation of BRD4 using the optimized degrader molecule dBET6 led to a global breakdown of transcription, therefore having more rapid and dramatic consequences in cancer cells [236]. The partial selectivity of BRD4-inhibtion for cancer specific enhancer and super-enhancer programs was not observed with this BRD4 degrader. This data demonstrated, that indeed BRD4 is a common essential gene with a non-redundant function in transcriptional elongation in malignant as well as normal cells. Therefore, the degradation of BRD4 has detrimental consequences, indicating a clear mechanistic difference to bromodomain-inhibition. Presumably, these differences are due to a more rapid and profound disruption of the transcription machinery at actively transcribed genes. Even though it might be challenging to manage toxicity of this type of treatment in more advanced pre-clinical stages of development, BET-degradation by dBET6 has been shown to be an efficient and tolerable treatment strategy in a mouse model of glioblastoma, indicating a potential therapeutic window in some transcriptionally driven cancers [237]. Nevertheless, based on the current data, it is questionable if BRD4 degradation is a viable therapeutic approach for targeted therapy rather than a cytotoxic treatment with a therapeutic window in aggressive cancers, similar to chemotherapy. However, these data clearly demonstrate different functional consequences when one uses small molecules to inhibit a protein’s function as compared to complete eradication of the protein.

**Figure 6:**
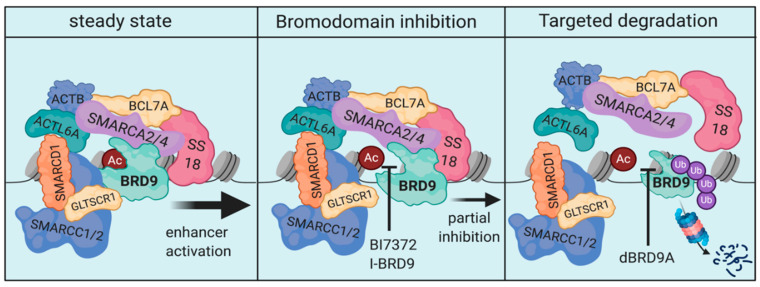
Schematic of the ncBAF-complex and the molecular consequences of BRD9-inhibition and BRD9-degradation. The chromatin remodeling function of the ncBAF complex is essential in maintenance of SS18-fusion driven synovial sarcoma (**left**). BRD9-bromodomain-inhibition leads to only a partial dissociation of the complex limiting cellular efficacy (**middle**). Targeted degradation of BRD9, on the other hand, de-stabilizes the complex to a greater extent, enabling increased cellular efficacy (**right**).

A clearer example for the potential therapeutic advantages of bromodomain-protein degradation are the pre-clinical observations made in synovial sarcoma regarding targeting of BRD9. As opposed to BRD4, BRD9 is a selective dependency in certain cancer types, including AML and synovial sarcoma [238,239,240]. BRD9 is a part of the mammalian SWI/SNF chromatin remodeling complex, more specifically the ncBAF complex [241,242]. Dysfunctional BAF-complexes have a specific relevance in synovial sarcoma, since the disease is driven by SS18-SSX fusion oncogenes. SS18 itself is a part of mammalian BAF-complexes, therefore, its involvement in oncogenic fusions in synovial sarcoma drives aberrant SWI/SNF function. Importantly, genetic targeting of BRD9 using different single-guide RNAs was detrimental for synovial sarcoma cells. A selective inhibitor of the BRD9-bromodomain on the other hand showed only modest cellular activity at reasonable drug concentrations. ChIP-sequencing demonstrated that BRD9-bromodomain inhibition only led to a sub-complete dissociation of BRD9 from chromatin (Figure 6). When utilizing a PROTAC of BRD9 (dBRD9A), the therapeutic efficacy in synovial sarcoma could be substantially improved due to elimination of BRD9 and a suspected higher degree of ncBAF complex disruption [238]. These findings have prompted the development of orally bioavailable degraders of BRD9. The first clinical trial investigating one of these compounds in synovial sarcoma are projected to start by the end of 2021.

## 5. Conclusions

Targeting epigenetic modulators for cancer therapy is a highly dynamic field that has led to the pre-clinical and clinical establishment of several molecules during the last decade. A major limitation in this process is the fact that a number of compounds, including most enzymatic inhibitors of epigenetic writers and erasers, have only modest single-agent activity. One opportunity to overcome this limitation is the establishment of efficacious combination therapy regimens. Another important consideration is that most enzymatic inhibitors have only limited potential to disrupt the integrity of chromatin-bound multiprotein complexes, potentially allowing for adaptation and resistance development by recruitment of alternative effectors. The evolving development of inhibitors of protein–protein interactions and targeted protein degraders offers an exciting opportunity to overcome these issues. It has been shown that the degree of physical disruption of certain oncogenic programs is significantly improved using these targeting approaches. Furthermore, proteins without a tractable enzymatic pocket can now be targeted using small molecules. With Menin-MLL1-interaction inhibitors the first class of compounds disrupting epigenetic protein–protein interactions recently entered phase 1 clinical trials. Beyond this, a number of compounds that aim to target previously “undruggable” proteins are now in preclinical and clinical pipelines and will significantly expand our toolbox for pharmacologic interrogation of aberrant epigenetic programs in myeloid malignancies and solid cancers in the years to come.

## Figures and Tables

**Table 1:** Clinical development stage of histone methyltransferase enzymatic inhibitors.

Drug	Target	Clinical Phase	Indication
Tazemetostat (EPZ-6438)	EZH2	approved	Rhabdoid tumors, Follicular lymphoma
Tazemetostat (EPZ-6438)	EZH2	phase 2	Diffuse Large B-Cell Lymphoma, Prostate Cancer, Synovial Sarcoma, Epitheloid Sarcoma, Mesothelioma, Squamous-cell Carcinoma, Urothelial Carcinoma, Ovarian and Endometrial Carcinoma, Melanoma,
GSK2816126	EZH2	phase 1	Diffuse Large B Cell Lymphoma, Follicular Lymphoma, Other Non-Hodgkin’s Lymphomas, Solid Tumors, Multiple Myeloma
CPI-1202	EZH2	phase 2	Diffuse Large B-Cell Lymphoma, Prostate Cancer, Advanced Solid Tumors
Pinometostat (EPZ-5676)	DOT1L	phase 2	Acute Myeloid Leukemia, Acute Lymphatic Leukemia
Tranylcypromine	LSD1	phase 2	Acute Myeloid Leukemia, Myelodysplastic Syndrome
Iadademstat (ORY-2001)	LSD1	phase 2	Alzheimer’s Disease
Bomedemstat (IMG-7289)	LSD1	phase 2	Essential Thrombocythemia, Polycythemia vera, Myelofibrosis, Acute Myeloid Leukemia, Myelodysplastic Syndrome

Summary of drugs discussed in this manuscript with their target, clinical stage, and indications.
